# Prophylactic and Therapeutic Effects of Oral Immunotherapy on Birch Pollen-Induced Allergic Conjunctivitis in Mice with a Rice-Based Edible Vaccine Expressing a Hypoallergenic Birch Pollen Allergen

**DOI:** 10.3390/cells10123361

**Published:** 2021-11-30

**Authors:** Waka Ishida, Tatsuma Kishimoto, Fumio Takaiwa, Ken Fukuda

**Affiliations:** 1 Department of Ophthalmology and Visual Science, Kochi Medical School, Kochi University, Kochi 783-8505, Japan; wakai@kochi-u.ac.jp (W.I.); t.kishimoto@kochi-u.ac.jp (T.K.); 2 Institute of Agrobiological Sciences, National Agriculture and Food Research Organization, Ibaraki 305-8602, Japan; takaiwa@affrc.go.jp

**Keywords:** immunotherapy, transgenic rice, allergic conjunctivitis, birch pollen, cytokines, regulatory T cells

## Abstract

We investigated the prophylactic and therapeutic effects of the oral administration of transgenic rice seeds expressing a hypoallergenic Bet v 1 derivative of allergic birch pollen conjunctivitis in mice. Transgenic rice seed depositing a chimeric molecule called TPC7 (tree pollen chimera 7) created by DNA shuffling of Bet v 1 family sequences from birch, alder and hazel in protein bodies of endosperm was generated. BALB/c mice were sensitized to birch pollen in alum and challenged with pollen in eyedrops. They were fed TPC7 transgenic or non-transgenic (control) rice seeds for 14 d before sensitization (prophylactic protocol) or 17 d after sensitization (therapeutic protocol). The clinical score and number of conjunctival eosinophils were significantly lower in TPC7-fed mice than in the control mice based on both the prophylactic and therapeutic protocols. Serum concentration of allergen-specific IgE did not differ between TPC7-fed and control groups in either protocol. Prophylactic administration of TPC7 downregulated the production of IL-4 and IFN-γ, whereas therapeutic administration of TPC7 upregulated the production of IFN-γ by allergen-stimulated splenocytes. Prophylactic or therapeutic oral administration of transgenic rice expressing TPC7 suppressed birch pollen-induced allergic conjunctivitis in mice. Feeding transgenic rice is a potentially effective approach as an allergen-specific immunotherapy for allergic conjunctivitis.

## 1. Introduction

The prevalence of allergic diseases, including conjunctivitis, rhinitis, asthma, and atopic dermatitis, is rapidly increasing worldwide [1,2]. Allergic conjunctivitis is an inflammatory condition of the conjunctiva triggered by exposure to sensitized allergens, such as pollen, animal dander, house dust mite, and other environmental antigens [3]. Pollinosis or hay fever is one of the most common allergic diseases, causing both nasal and ocular symptoms. Birch pollen allergy is one of the most prevalent allergic diseases in North America, northern Europe, Russia, China, and northern Japan [4,5,6,7]. The main therapeutic modality for allergic conjunctivitis is eye drops with antihistamines, mast cell stabilizers, and/or corticosteroids, regardless of the sensitized allergens. Although these medications can temporarily reduce clinical symptoms, they do not constitute a permanent cure. Allergen-specific immunotherapy is the only disease-modifying approach to suppress the symptoms of allergic diseases over the long term [8,9]. Allergen-specific immunotherapy is performed by the repeated administration of antigens via several different routes, including subcutaneous injections (subcutaneous immunotherapy: SCIT), sublingual application (sublingual immunotherapy: SLIT), and oral administration (oral immunotherapy: OIT). Successful treatment with allergen-specific immunotherapy has been reported in the past century for allergic rhinitis, conjunctivitis, and asthma [9,10,11]. Among immunotherapies, sublingual immunotherapy is now widely used worldwide for pollinosis [12,13]. SLIT appears to be safer and more convenient than SCIT. However, given that SLIT takes several years of treatment to exert its effects, the low continuity rate is a major problem [14]. OIT has been investigated as an alternative approach for treating food allergies. Some clinical trials achieved successful desensitization of food allergies by OIT [15], and the FDA has approved peanut OIT for treating peanut-allergic children. The underlying mechanisms of OIT against food allergy remain unclear [16]. In addition, OIT in patients with pollinosis or allergic conjunctivitis has not been investigated.

In a previous study, we demonstrated that prophylactic and therapeutic oral administration of ovalbumin (OVA) suppressed OVA-induced experimental allergic conjunctivitis (EAC) in a mouse model, suggesting that OIT is also a candidate for radical treatment of allergic conjunctivitis [17]. For a more effective, convenient, and safer immunotherapy, we developed a rice-base edible vaccine expressing various antigens, including that of pollen or house dust mites [18,19,20]. Transgenic rice seeds have several benefits, such as their high production ability, stability at ambient temperature for several years, safety (no contamination with human pathogens), cost-effectiveness, and easy handling of oral administration. Particularly, antigens produced in rice seed permit oral administration, as bioencapsulated antigens are resistant to harsh gastric environments such as low pH and digestive enzymes [21]. Taking advantage of transgenic (Tg) rice seeds containing hypoallergenic modified antigens [22], we showed that the preventive and therapeutic effects of oral immunotherapy suppressed cedar pollen-induced EAC [23,24]. The safety and efficacy of OIT with Tg rice has been reported following oral administration to Japanese monkeys with Japanese cedar pollinosis. Healthy monkeys were not sensitized to Tg rice containing the hypoallergenic Japanese cedar pollen Cry j 1 and Cry j 2 allergens; in contrast, peripheral blood mononuclear cell proliferation and antigen-specific IgE levels were decreased in Japanese monkeys with Japanese cedar pollinosis [25].

Here, our goal was to investigate the prophylactic and therapeutic effects of oral administration of Tg rice seeds expressing hypoallergenic birch pollen allergen on induced birch pollen allergic conjunctivitis using a mouse model.

## 2. Materials and Methods

### 2.1. Mice

BALB/c mice were purchased from Japan SLC (Hamamatsu, Shizuoka, Japan) and maintained under specific pathogen-free conditions at the animal facility of Kochi Medical School. Female mice (eight weeks old) were used in the experiments.

### 2.2. Transgenic Rice

Tg rice expressing tree pollen chimera 7 (TPC7) or native Bet v 1 was generated as described previously [26,27]. TPC7 is a hypoallergenic Bet v 1 tolerogen against birch pollen allergy, selected by DNA shuffling of 14 types of Fagales tree pollen allergens [28]. A homogeneous line of Tg rice strain that accumulates the TPC7 as an oral vaccine at the level of about 207 µg/grain was selected and propagated under natural light conditions in an enclosed glass greenhouse [27]. Mature seeds were harvested and then dehulled and pulverized. The TPC7 was deposited in giant spherical endoplasmic reticulum-derived protein bodies in endosperm cells.

### 2.3. Feeding and Sensitization of Mice

**Figure 1:**
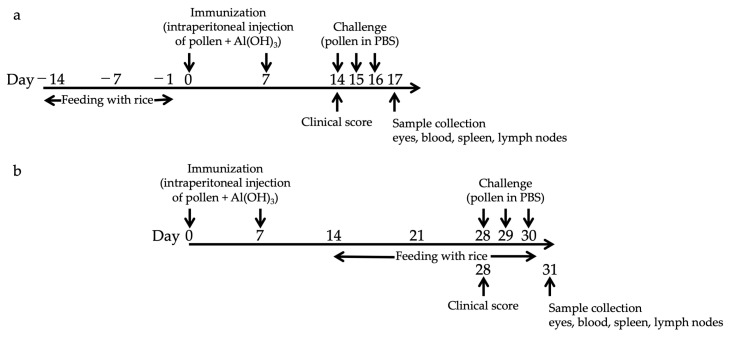
Schematic representation for the protocol of oral administration with Tg rice seed and induction of experimental allergic conjunctivitis. Mice were fed Tg or non-Tg control rice seed either orally ad libitum for 14 d before sensitization (prophylactic protocol, (**a**)) or 17 d after sensitization (therapeutic protocol, (**b**)). The Tg rice fed group was administered 3 g non-Tg rice seed containing 0.2 g Tg rice seed per day. The control group was fed 3 g non-Tg rice seed per day. To induce EAC, 0.1 mg of birch pollen was mixed with 1.3 mg of alum and intraperitoneally injected twice at an interval of 7 d. Seven days (prophylactic protocol, (**a**)) or 21 d (therapeutic protocol, (**b**)) after the second sensitization, birch pollen in PBS (1.2 mg per 4 μL per eye) was administered onto the eyes of the mice for three consecutive days.

Mice were fed Tg or non-transgenic (non-Tg) rice seeds orally ad libitum for either 14 d before sensitization (prophylactic protocol) or 17 d after sensitization (therapeutic protocol). The Tg rice fed group was administered fine powder of 3 g non-Tg rice seed containing 0.2 g TPC7 Tg rice seed or 0.4 g Bet v 1 Tg rice seed per day. Each mouse was estimated to consume approximately 2.7 mg of TPC7 antigens daily. The control group was fed a fine powder of 3 g of non-Tg rice seeds per day. The mice were allowed access to normal food and drinking water ad libitum. Birch pollen-induced EAC was performed as described previously with slight modifications [23]. Briefly, birch pollen (0.1 mg) (Biostir, Hyogo, Japan) was mixed with 1.3 mg of alum (Sigma-Aldrich, St. Louis, MO, USA) and intraperitoneally injected twice at intervals of 7 d. Either 7 d (prophylactic protocol) or 21 d (therapeutic protocol) after the second sensitization, birch pollen in PBS (1.2 mg per 4 μL per eye) was administered to the eyes of the mice for three consecutive days (Figure 1). Clinical symptoms were evaluated at 20 min after the third challenge. At 24 h after the last challenge, the conjunctiva, spleen, blood, and submandibular lymph node were isolated for histological analysis, cytokine production assays, measurement of serum immunoglobulin E (IgE), and flow cytometric analysis, respectively.

### 2.4. Evaluation of Clinical Conjunctival Early Phase Reaction

Clinical symptoms such as tearing, lid edema, chemosis, and conjunctival redness were graded on a 0–3 scale 20 min after the challenge, and the clinical score was expressed as the total score, consisting of the sum of each of these four categories as described previously [29].

### 2.5. Histological Evaluation of Conjunctiva

Histological analysis was performed to evaluate infiltrated eosinophils in the conjunctiva, as described previously [30]. Briefly, the eyes were fixed in 10% buffered formalin. Paraffin sections were cut to 2 μm and stained with Giemsa solution. Infiltrating eosinophils in the conjunctiva were counted by observer masking.

### 2.6. Measurement of Total and Bet v 1 Specific-IgE

Tolal IgE in the serum was evaluated using ELISA, as described previously [23]. Briefly, 96-well plates were coated overnight at 4 °C with anti-mouse IgE antibody (2 μg/mL; Southern Biotechnology, Birmingham, AL, USA). The plates were washed and incubated with blocking buffer (1% bovine serum albumin in PBS) for 3 h at room temperature. Samples or IgE standard (TNP-KLH-specific IgE; BD Biosciences, Fanklin Lakes, NJ, USA) were added to each well and incubated for 2 h at room temperature. The plates were then washed, and biotin-conjugated anti-mouse IgE (BD Biosciences) was added to each well for 1 h at room temperature and washed before adding avidin-conjugated alkaline phosphatase (Sigma-Aldrich) for 1 h. After washing the plates, *p*-nitrophenyl phosphate substrate (Sigma-Aldrich) was added to each well, the plates were incubated for 15 min at room temperature, and absorbance was measured at 405 nm. The allergen (Bet v 1)-specific IgE was measured by ELISA, slightly modified from a previously described method [31]. The 96-well EIA plates were coated overnight at 4 °C with 2 μg/mL anti-mouse IgE antibodies (Southern Biotechnology, Birmingham, AL, USA). After washing and blocking the plates with Blockace (DS Pharma Biomedical, Osaka, Japan), samples were added to each well, which was then washed. To detect Bet v 1-specific antibodies, total protein extracts of birch pollen (GREER, Lenoir, NC) were biotinylated using an EZ-Link Sulfo-NHS-LC-Biotinylation kit (Thermo Fisher Scientific, Waltham, MA, USA) according to the manufacturer’s procedure and added to the wells as secondary antibodies. After washing the plates, streptavidin-conjugated horseradish peroxidase (Thermo Fisher Scientific) was added to each well. The reaction was developed with 100 μL of the substrate solution (R&D Systems, Minneapolis, MN, USA) and terminated by the addition of 50 μL of 1 M H_2_SO_4_. The absorbance of each well was determined at a wavelength correction for 450 nm at 570 nm with a microplate reader. Given that a Bet v 1-specific IgE standard was not available, the results were expressed as absorbance units.

### 2.7. Mesurement of Cytokine Release by Splenocytes

Splenocytes were incubated for 48 h at 1 × 10^7^ cells/mL with Bet v 1 extract (GREER) at a concentration of 20 μg/mL in RPMI 1640 medium (Wako Pure Chemical Industries, Osaka, Japan) supplemented with 20% fetal bovine serum (Thermo Fisher Scientific), 0.2 mM sodium pyruvate (Sigma-Aldrich) and 0.1 mM 2-mercaptoethanol (Nacalai Tesque, Kyoto, Japan). The concentrations of interleukin (IL)-4 and interferon-γ (IFN)-γ in the culture supernatants were then measured using a Bioplex system (Bio-Rad, Hercules, CA, USA) according to the manufacturer’s protocol [23]. Data were expressed as the mean ± SEM (pg/mL).

### 2.8. Flow Cytometric Analysis of CD4+CD25+Foxp3+ Expression by Lymphocytes from Lymph Nodes and the Spleen

Flow cytometric analysis of CD4+CD25+Foxp3+ expression in lymphocytes from the mesenteric lymph nodes and spleen was performed, as described previously [24]. Briefly, the cells were washed with flow cytometry staining buffer (FSB, PBS containing 1% fetal bovine serum and 0.1% sodium azide) and incubated for 30 min on ice with LIVE/DEAD Fixable Dead Cell Stain Kit (Thermo Fisher Scientific), washed with FSB, and fixed for 25 min on ice with Foxp3 staining buffer (Thermo Fisher Scientific). The cells were washed with permeabilization buffer (Thermo Fisher Scientific) and stained for 25 min on ice with fluorescein isothiocyanate-labeled antibodies to CD4, phycoerythrin-labeled antibodies to CD25, and phycoerythrin-Cy5-labeled antibodies to Foxp3 (Thermo Fisher Scientific). The cells were washed with FSB, and stained cells were measured using an Attune flow cytometer (Thermo Fisher Scientific). The percentage of CD25+Foxp3+ cells was analyzed by gating the CD4+ population.

### 2.9. Statistical Analysis

The transgenic or control rice-fed groups were compared regarding clinical score, serum Ig levels, cytokine production concentration in the supernatants, and expression of CD4+CD25+Foxp3+ cells using an unpaired Student’s *t* test. The Mann–Whitney U test was used to assess the infiltration of eosinophils. Statistical significance was set at *p* < 0.05.

## 3. Results

### 3.1. Prophylactic Administration with Tg Rice Suppresses the Development of EAC

**Figure 2:**
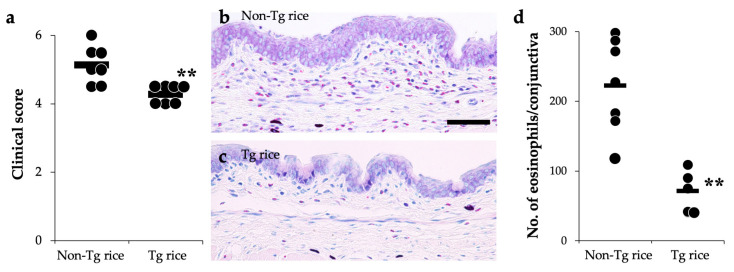
Prophylactic administration with Tg rice suppresses the development of allergic conjunctivitis. Mice were fed with Tg rice seed or control (non-Tg) rice seed ad libitum for 14 d before the first sensitization with birch pollen. At 20 min after the last birch pollen challenge, clinical appearance was evaluated. ** *p* < 0.01, Tg vs. non-Tg rice-fed group according to an unpaired Student’s *t* test (seven mice per group) (**a**). At 24 h after the third challenge, the eyes were isolated for histological analysis according to the number of eosinophils in the conjunctiva (**b**–**d**). ** *p* < 0.01, Tg vs. non-Tg rice-fed group according to the Mann–Whitney U test (non-Tg rice group: seven mice per group and Tg rice group: five mice per group). The dots and bars within (**a**,**d**) indicate mean values for the two eyes of each mouse and the overall median values, respectively.

**Figure 3:**
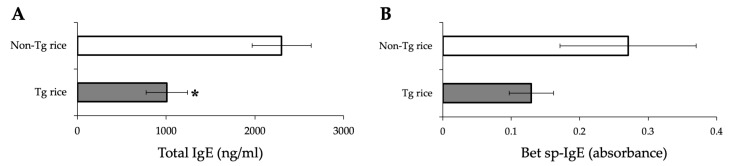
Serum concentration of total IgE (**A**) and Bet v 1-specific IgE (**B**) in mice fed Tg or non-Tg rice. Data are presented as ng/mL or absorbance units. * *p* < 0.05, Tg vs. non-Tg rice-fed group according to the unpaired Student’s *t* test (three mice per group).

**Figure 4:**
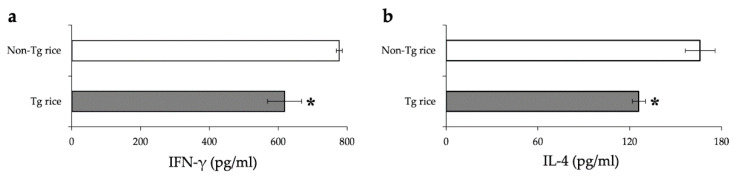
Release of IFN-γ (**a**) and IL-4 (**b**) by the antigen-stimulated splenocytes from mice fed Tg or non-Tg rice. Data are the means ± SEM. * *p* < 0.05, Tg vs. non-Tg rice-fed group according to unpaired Student’s *t* test (three mice per group).

We first examined the effects of oral administration of Tg rice seeds on the prophylactic protocol (Figure 1a). Mice were fed Tg rice seeds or control rice seeds orally ad libitum for 14 d before the first sensitization. The clinical score (Figure 2a) and number of eosinophils in the conjunctiva (Figure 2b–d) were significantly lower in the Tg rice-fed group than in the control group. In contrast, the serum concentration of Bet v 1-specific IgE levels did not significantly differ between the two groups (Figure 3). The Tg rice-fed group also significantly suppressed the allergen-induced production of both IL-4 and IFN-γ by isolated splenocytes (Figure 4). The expression of CD4+CD25+Foxp3+ cells was not significantly different between the groups in isolated lymphocytes from mesenteric lymph nodes and spleens (Table 1).

### 3.2. Therapeutic Administration with Tg Rice Suppresses the Established EAC

**Figure 5:**
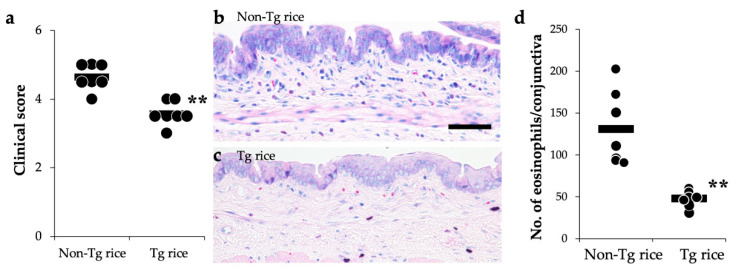
Therapeutic administration with Tg rice suppresses the development of EAC. Mice were fed Tg rice seed or control (non-Tg) rice seed ad libitum for 17 d after the sensitization with birch pollen. At 20 min after the last birch pollen challenge, clinical appearances were evaluated. ** *p* < 0.01, Tg vs. Non-Tg rice-fed group according to unpaired Student’s *t* test (seven mice per group) (**a**). At 24 h after the third challenge, the eyes were isolated for histological analysis of the number of eosinophils in the conjunctiva (**b**–**d**). ** *p* < 0.01, Tg vs. Non-Tg rice-fed group according to the Mann–Whitney U test (seven mice per group). The dots and bars within (**a**,**d**) indicate mean values for the two eyes of each mouse and the overall median values, respectively.

**Figure 6:**
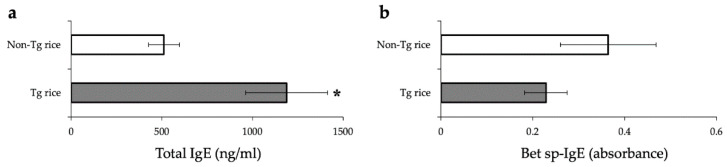
Serum concentration of total IgE (**a**) and Bet v 1-specific IgE (**b**) in mice fed with Tg or non-Tg rice. Data are presented as ng/mL or absorbance units. * *p* < 0.05, Tg vs. non-Tg rice-fed group according to unpaired Student’s *t* test (three mice per group).

**Figure 7:**
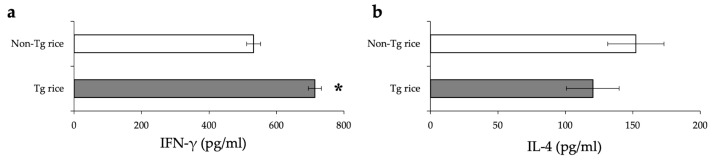
The release of IFN-γ (**a**) and IL-4 (**b**) by the antigen-stimulated splenocytes from mice fed with Tg or non-Tg rice. Data are means ± SEM. * *p* < 0.05, Tg vs. non-Tg rice-fed group according to unpaired Student’s *t* test (three mice per group).

To examine whether therapeutic administration of Tg rice can suppress established EAC, mice were fed with Tg or control rice seeds ad libitum for 17 d from 7 d after the second sensitization (Figure 1b). The clinical symptoms (Figure 5a) and number of eosinophils in the conjunctiva (Figure 5b–d) were significantly lower in the Tg rice-fed group than in the control group. The serum concentration of Bet v 1-specific IgE levels did not differ significantly between the groups (Figure 6). The Tg rice-fed group showed significantly upregulated allergen-induced production of IFN-γ, but not IL-4, in isolated splenocytes (Figure 7). The expression of CD4+CD25+Foxp3+ cells was not significantly different between the two groups in isolated lymphocytes from mesenteric lymph nodes or spleens (Table 2).

### 3.3. Effects of Prophylactic and Therapeutic Oral Administration of Native Bet v 1-Expressing Tg Rice on EAC

**Figure 8:**
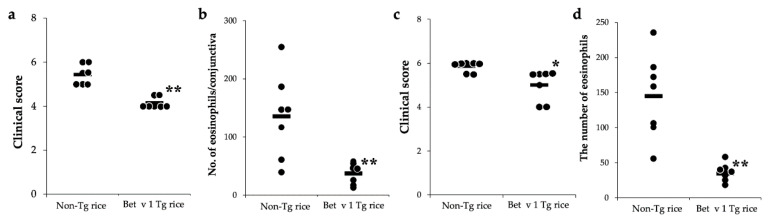
Prophylactic and therapeutic administration with Tg rice expressing Bet v 1 suppresses EAC development. Mice were fed Bet v 1 Tg rice seed or control (non-Tg) rice seed ad libitum for 14 d before the first sensitization ((**a**,**b**) prophylactic administration) or for 17 d after the sensitization ((**c**,**d**), therapeutic administration) with birch pollen. At 20 min after the last birch pollen challenge, clinical appearances were evaluated. * *p* < 0.05, ** *p* < 0.01, Tg vs. non-Tg rice-fed group according to unpaired Student’s *t* test (seven female mice per group) (**a**,**c**). At 24 h after the third challenge, the eyes were isolated for histological analysis of the number of eosinophils in the conjunctiva (**b**,**d**). ** *p* < 0.01, Tg vs. non-Tg rice-fed group according to the Mann–Whitney U test (seven female mice per group). The dots and bars indicate the mean values for the two eyes of each mouse and the overall median values, respectively.

We also examined the effects of oral administration of Tg rice expressing native Bet v 1 on allergic conjunctivitis. As shown in Figure 8, the administration of Tg rice significantly suppressed clinical symptoms and eosinophil infiltration into the conjunctiva in both the prophylactic and therapeutic settings.

**Figure 9:**
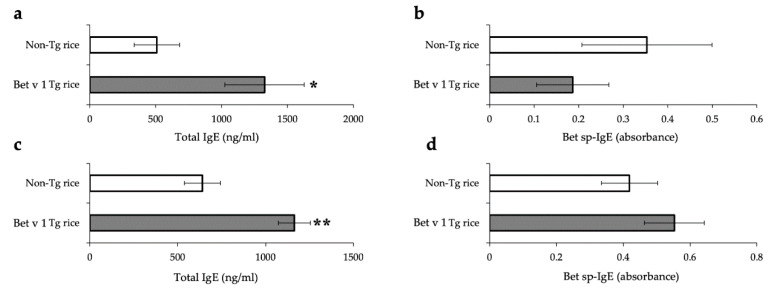
Serum concentration of total IgE (**a**,**c**) and Bet v 1-specific IgE (**b**,**d**) in mice fed with Bet v 1 Tg or non-Tg rice in prophylactic (**a**,**b**) and therapeutic (**c**,**d**) protocol. Data are presented as ng/mL or absorbance units. * *p* < 0.05, ** *p* < 0.01, Bet v 1 Tg vs. non-Tg rice-fed group according to unpaired Student’s *t* test (three female mice per group).

For both the prophylactic and therapeutic protocols, the total IgE serum concentration was significantly increased in the Bet v 1 Tg rice-fed group compared to in the control group (Figure 9). Bet v1-specific IgE levels did not differ significantly between the two groups. The production of IFN-γ and IL-4 in antigen-specific recall responses was not significantly different between the two groups (data not shown). Although the expression of CD4+CD25+Foxp3+ cells did not significantly differ between the two groups in the isolated lymphocytes from mesenteric lymph nodes or spleens in the prophylactic protocol, the Bet v 1 Tg rice-fed group showed significantly upregulated expression of CD4+CD25+Foxp3+ cells from the mesenteric lymph nodes, but not from the spleens in the therapeutic protocol (Table 3).

## 4. Discussion

In this study, we demonstrated that feeding with Tg rice seeds expressing hypoallergenic modified birch pollen antigen TPC7 had both prophylactic and therapeutic effects on allergic conjunctivitis in a mouse model of birch pollen allergic conjunctivitis. Given that this antigen (TPC7), generated by DNA shuffling, was demonstrated to have lower allergenicity and higher immunogenicity than native Bet v 1 [28], OIT with this Tg rice could be a more effective and safer therapeutic modality. Reduced risk of anaphylaxis could allow high-dose administration, which would shorten the treatment period.

More than 90% of patients with birch pollinosis are reportedly sensitized to Bet v 1. The antigen expressed in rice seed used in this study was the TPC7 hypoallergenic tolerogen [28], and approximately 207 µg/grain was accumulated at the highest levels [27]. TPC7 was generated by in vitro random recombination with the use of DNA shuffling, resulting in the reduction of IgE binding capacity thanks to the modified three-dimensional structure. In addition to the reduced risk of anaphylaxis, this modified antigen also reportedly exhibited higher immunogenicity than native Bet v 1 [28]. Strong IgE cross-reactivity between Bet v 1 and other pollen allergens of Fagales trees, including hazelnut (Cor a 1), oak (Que a 1), alder (Aln g 1), and hornbeam (Car b 1), have been reported [32,33,34,35]. Additionally, Bet v 1-specific IgE also cross-reacts with food allergens in fruits and vegetables, including apple Mal d 1, soybean Gly m 4, carrot Dau c 1, and peanut Aha h 8, and causes oral allergy syndrome [36]. A retrospective study in Finland has shown that more than 80% of patients sensitized to birch pollen were also sensitized to hazelnut [7]. Therefore, OIT with this Tg rice could be effective not only for birch pollen allergy but also for other allergic diseases caused by these antigens.

The underlying mechanisms of tolerance by allergen-specific immunotherapy are not fully understood, but various cellular and humoral responses could be involved, such as alteration of T cell and B cell responses to allergens, activation of Treg cells, reduction of allergen-specific IgE, or increase of IgG4 [37]. These mechanisms may depend on various factors, such as the dose, duration, timing, frequency, specificity, and route of administration of allergens used as tolerogens [17]. We have demonstrated the effects of OIT in transgenic rice expressing hypoallergenic Japanese cedar pollen allergens for cedar pollen-induced allergic conjunctivitis in mice. Although both prophylactic [23] and therapeutic [24] oral administration of Tg rice suppressed allergic conjunctivitis, the underlying mechanisms were different. In terms of antigen-induced cytokine release by splenocytes, prophylactic administration suppressed both Th1 and Th2 cytokines, whereas therapeutic administration increased Th1 cytokines. Serum levels of antigen-specific IgE were significantly decreased by prophylactic administration, but not by therapeutic administration. In this study using Tg rice seed expressing TPC7, systemic immunological reactions similar to those in these previous studies were observed. Prophylactic protocol feeding suppressed both IL-4 and IFN-γ production (Figure 4), whereas therapeutic protocol feeding increased the production of IFN-γ, but not IL-4 (Figure 7). The suppressive effects of conjunctivitis could be mediated by the induction of T cell anergy or deletion by prophylactic feeding, whereas the shift from Th2 to Th1 occurred by therapeutic feeding. A Th2 to Th1 shift or an early increase in IFN-γ production was reportedly associated with clinical improvement of symptoms in patients treated with SCIT or SLIT [38,39,40,41,42]. Infiltration of eosinophils into the conjunctiva is reportedly regulated by Th1/Th2 cytokines; IL-4 induces eosinophil infiltration into the conjunctiva, and IFN-γ counteracts this effect of IL-4 [43]. Therefore, the increase in IFN-γ with therapeutic administration of Tg rice may have contributed to suppressing EAC in this study. Although allergen-specific Treg cells are among the important cells inducing tolerance, the number of CD4+CD25+Foxp3+ Treg cells in mesenteric lymph nodes and spleens was not significantly different between the non-Tg and Tg rice-fed groups in both of the current prophylactic and therapeutic protocols. Serum antigen-specific IgG4 is increased after starting OIT [16], and IgG4 from patients treated with OIT can suppress antigen-stimulated activation of mast cells and basophils [44]. We did not measure the levels of antigen-specific IgG4, and the role of antigen-specific IgG4 in mice treated with Tg rice should be further clarified.

We also examined the effects of OIT with Tg rice expressing native Bet v 1. Oral administration of native Bet v 1 Tg rice significantly suppressed clinical symptoms and eosinophil infiltration into the conjunctiva, in a similar manner to Tg rice expression of hypoallergenic TPC7. However, the immunological reactions, including serum total IgE levels, cytokine production by antigen-stimulated splenocytes, and number of Treg cells in mesenteric lymph nodes, were different in mice administered Tg rice. Further investigation of the immunological mechanisms elicited by hypoallergenic antigens and native antigens is needed for safe clinical application.

Immunotherapy can be performed via various routes of administration, including SCIT, SLIT, and OIT. Recently, intralymphatic and epicutaneous immunotherapies have been developed, and their clinical effects are being evaluated [45,46]. Each administration route has advantages and disadvantages [37]. OIT with commonly consumed food is more convenient than SIT. Because rice is a staple food in many Asian regions, OIT with Tg rice seed may increase treatment adherence in these populations. One of the disadvantages of OIT is the degradation of antigens by gastric digestive enzymes. TCP7 and Bet v 1 were deposited in the endoplasmic reticulum-derived protein bodies of rice endosperm cells. Therefore, when Tg rice seeds are orally administered, these antigens are highly resistant to digestive enzymes, temperature, and the harsh conditions of the gut, since they are bio-encapsulated by the double barriers of protein bodies and cell walls characteristic of plant cells. Therefore, by cooking and eating Tg rice as regular rice, the modified antigens are effectively delivered to the gut-associated lymphoid tissues.

## 5. Conclusions

Oral immunotherapy with edible transgenic rice expressing a hypoallergenic Bet v 1 tolerogen could be a safe, effective, and convenient novel immunotherapy for birch pollen allergy.

## Figures and Tables

**Table 1:** Number of CD4+CD25+Foxp3+ Treg cells in mice fed Tg rice or non-Tg rice in mesenteric lymph nodes and spleens.

	Non-Tg Rice	Tg Rice
Mesenteric lymph node	4.82 ± 0.36	4.32 ± 0.12
Spleen	2.26 ± 0.21	1.88 ± 0.07

Data represent the percentage of CD4+CD25+Foxp3+ cells among all CD4+ cells and are the mean ± SEM (three mice per group).

**Table 2:** Number of CD4+CD25+Foxp3+ Treg cells in mice fed Tg rice or non-Tg rice.

	Non-Tg Rice	Tg Rice
Mesenteric lymph node	4.50 ± 0.24	4.43 ± 0.36
Spleen	2.39 ± 0.07	2.25 ± 0.13

Data represent the percentage of CD4+CD25+Foxp3+ cells among all CD4+ cells and are the mean ± SEM (three mice per group).

**Table 3:** Number of CD4+CD25+Foxp3+ Treg cells in mice fed Tg rice expressing native Bet v 1 or non-Tg rice.

	Prophylactic Administration		Therapeutic Administration	
	Non-Tg Rice	Bet v 1 Tg Rice	Non-Tg Rice	Bet v 1 Tg Rice
Mesenteric lymph node	4.97 ± 0.10	4.62 ± 0.26	3.64 ± 0.16	5.23 ± 0.47 *
Spleen	2.53 ± 0.07	2.50 ± 0.25	2.52 ± 0.09	2.73 ± 0.05

Data represent the percentage of CD4+CD25+Foxp3+ cells among all CD4+ cells and are the mean ± SEM (three female mice per group). * *p* < 0.05, Tg vs. non-Tg rice-fed group according to unpaired Student’s *t* test (three mice per group).
